# Coordinated dynamics of circulating neutrophils, regulatory T lymphocytes, myeloid derived suppressor cells and distinct granulocytic subsets reveal a complex immune network in metastatic colorectal cancer

**DOI:** 10.3389/fonc.2026.1873352

**Published:** 2026-08-04

**Authors:** Zafeiris Zafeiriou, Anna Koutoulaki, Despoina Aggouraki, Filippos Koinis, Nikolaos Vardakis, Alexandra Voutsina, Katerina Papadogiannaki, Konstantia Kotta, Eleni-Kyriaki Vetsika, Dimitris Mavroudis, Vassilis Georgoulias, Athanasios Kotsakis

**Affiliations:** 1 Department of Medical Oncology, School of Medicine, University of Crete, Heraklion, Greece; 2 Laboratory of Tumor Cell Biology, School of Medicine, University of Crete, Heraklion, Greece; 3 Department of Medical Oncology, School of Medicine, University of Thessaly, Larissa, Greece; 4 Institute of Chemical Biology, National Hellenic Research Foundation (NHRF), Athens, Greece; 5 Institute of Applied Biosciences, Centre for Research and Technology, Thessaloniki, Greece; 6 Department of Basic and Clinical Sciences, Medical School, University of Nicosia, UNIC Athens, Athens, Greece

**Keywords:** colon cancer, granulocytic, HLA-DR, lymphocytes, myeloid derived suppressor cells, neutrophils, NLR, regulatory T lymphocytes

## Abstract

**Introduction:**

Circulating immune cells serve as readily accessible biomarkers in cancer patients. Some of them, such as neutrophils, lymphocytes as well as the neutrophil to lymphocyte ratio (NLR), have been validated as prognostic indicators of disease outcome whereas the significance of others remains controversial due to conflicting findings. Moreover, the interplay and correlation among these markers have not been thoroughly investigated nor has their dynamic course during chemotherapy.

**Methods:**

Blood was collected from 16 healthy donors and 90 patients receiving first line chemotherapy for metastatic colorectal cancer before, during and after chemotherapy. Neutrophils, lymphocytes, circulating CD4^+^CD25^+^FOXP3^+^ regulatory T lymphocytes (Tregs), monocytic myeloid derived suppressor cells (mMDSCs), polymorphonuclear myeloid derived suppressor cells (pMDSCs) and other exploratory myeloid subsets were immunophenotyped and quantified with flow cytometry. Their frequencies were compared between healthy donors and patients and correlated with each other and with clinical parameters. Associations with progression-free and overall survival were evaluated using Cox proportional hazards regression and longitudinal changes in immune cell populations during chemotherapy were analyzed using linear mixed-effects models.

**Results:**

Circulating Tregs were significantly reduced in patients with high neutrophil counts (7% vs 17%, p=0.04), increased significantly during treatment in responders, particularly in patients achieving complete or partial response (+16%, *p* = 0.003) but did not demonstrate prognostic significance. pMDSCs were further stratified by CD16 expression into a CD16^+^ subset associated with favorable outcome and a CD16^dim/−^ subset enriched in patients demonstrating poor prognosis. Frequency of circulating mMDSCs was modestly increased in patients but retained adverse prognostic significance (HR 1.98, CI 1.04-3.78, *p=*0.039) and correlated with the CD16^dim/−^ pMDSC fraction. Mature CD15^+^CD33^-^ polymorphonuclear cells were significantly expanded in patients (79% vs 73%, *p=*0.003) and correlated with adverse clinical features, whereas lower levels correlated with improved survival (HR 0.54, CI 0.3-0.98, *p=*0.045). Rare HLA-DR^+^CD16^+^ granulocytic subset demonstrated significantly reduced levels in patients compared to healthy donors while their kinetics during treatment also correlated inversely with disease burden.

**Discussion:**

Circulating immune cell subsets in metastatic colorectal cancer exhibit considerable baseline heterogeneity and distinct immune kinetics during chemotherapy. Our findings demonstrate that conventional leukocyte populations, when evaluated individually, together with detailed immunophenotyping provide complementary prognostic information, revealing previously unappreciated heterogeneity within major immune cell populations and a network of novel interrelationships among circulating immune cells. Together, these observations support comprehensive immune profiling as a promising approach for future biomarker development and immune-based patient stratification.

## 1 Introduction

Circulating immune cells in cancer patients have been recognized as a valuable source of prognostic information reflecting underlying immune cell involvement, altered haematopoiesis induced by tumor secreted cytokines and changes in immune cell trafficking. Neutrophilia and lymphopenia were early recognized in the peripheral blood of cancer patients and have each been independently associated with dismal prognosis (1, 2). The combination of both neutrophils and lymphocytes in a single fraction gave rise to the neutrophil to lymphocyte ratio (NLR) a commonly encountered biomarker thought to mirror an inflammatory state and associated with worse outcome in a number of malignant diseases and stages (3–5) but also in non malignant conditions (6).

The mechanistic explanation for lymphopenia’s association with adverse prognosis in diverse cancer types remains largely obscure and is thought to indicate a reduced tumoricidal immune capacity (7, 8). Neutrophils, on the other hand, can kill cancer cells in a number of ways; even more can they promote tumorigenesis (9). In cancer bearing hosts their maturation seems to diverse into pathological functional states able to suppress tumor directed T cell immunity (10). For these neutrophil forms the term Myeloid Derived Suppressor Cells (MDSCs) has been coined. No definite cell surface marker has been established for their unequivocal characterization by flow cytometry (11) and they are considered to either represent a subset of neutrophils or one of their differentiation states rather than a distinct cell population (12). However, diverse marker combinations have been considered acceptable for their characterization and have been widely used in order to quantify their presence and assess their prognostic significance in several solid malignancies, among which also colorectal cancer, where they portend a worse outcome (13). As a consensus, two main subsets have been recognized: polymorphonuclear MDSCs (pMDSCs), otherwise encountered as granulocytic MDSCs and monocytic MDSCs (mMDSCs). The minimum required phenotypical markers for their phenotypic characterization have been defined as CD11b+CD14-CD15+HLADRlow/- and CD11b+CD14+CD15-HLADRlow/-, respectively (11).

Regulatory T lymphocytes (Tregs) represent another population with the ability to suppress T cell function in the tumor microenvironment. Under physiological conditions Tregs are pivotal for self-tolerance by suppressing autoimmune reactions and immune hypersensitivity (14). In cancer patients they were found to be enriched in cancer tissues, draining lymph nodes and peripheral blood (15–17) where they are considered to act by suppressing tumor specific T cell immunity in the same way as they suppress autoimmunity. Consistently, Treg infiltrates predict a worse prognosis in some cancer types e.g. in ovarian, prostate and non-small cell lung cancer (18–20). There are cancer types (i.e. lymphoma, head and neck cancer and colorectal cancer), however, where the presence of cells with a Treg-phenotype in the tumor microenvironment (TME) has been found to correlate with favorable prognosis (21).

Colorectal cancer (CRC) is among the major causes of cancer related death worldwide. The treatment of metastatic disease includes a cytotoxic backbone in addition to agents targeting the VEGFR or the EGFR pathway. Immune checkpoint inhibitors, proven useful in other types of cancer, have efficacy only in the minority of mCRC cases with microsatellite instability (22)However, T-cell infiltrates in the TME are prevalent and possess prognostic significance comparable to the TNM classification (23) implying underlying immune evasion mechanisms that remain clinically unexploited.

Regarding circulating immune cells in CRC, increased neutrophils in the peripheral blood are a validated dismal prognostic marker in metastatic disease (24) and circulating Tregs (cTregs) have also been found to be expanded (15, 25) though reduced circulating Tregs numbers have also been reported (26). Treg infiltrates in CRC tissue correlate with favorable prognosis (27–29); in contrast CRC patients with lower Tregs in the circulation seem to fare better (30, 31). Furthermore, MDSCs are considered to be increased in the circulation of CRC patients where they are correlated with worse prognosis (32–34).

It is evident that the various immune cell populations present within the tumor microenvironment (TME) or in circulation may exhibit either redundant or complementary roles in terms of their functional activity and prognostic relevance. To date, the relationship between cTregs and circulating neutrophils, the - NLR, or circulating myeloid-derived suppressor cells (cMDSCs) in patients with metastatic CRC (mCRC) has not been elucidated. In the present study, cTregs, cMDSCs, neutrophils, lymphocytes, and additional exploratory myeloid subsets were quantified in patients with mCRC at baseline and throughout first-line chemotherapy. The aim of the current study was to evaluate their prognostic significance, characterize their dynamic changes during treatment, and assess their associations with patients’ clinical outcome.

## 2 Materials & methods

### 2.1 Patients and treatment

Patients with metastatic or locally advanced CRC, eligible to receive first line treatment, were enrolled in the study. Participating patients should not have received any prior chemotherapy, except in the adjuvant setting at least 3 months before enrollment and be older than 18 years. The type of treatment, FOLFOX or FOLFIRI with or without biological (bevacizumab or EGFR inhibitors according to KRAS/NRAS status), was chosen by the treating physician according to national guidelines. Response to treatment was assessed with CT scans as per RECIST 1.1 criteria after cycle 3 day 1, after cycle 6 day 15 or earlier if clinically indicated and subsequently as per treating physician’s choice. Absolute blood counts and other clinical parameters were extracted from patients’ records. The study has been conducted according to the principles of the Declaration of Helsinski, all patients gave their written informed consent and the study was approved by the local ethics and scientific committees of the University Hospital of Heraklion (decision number 3020/10-11-2010).

### 2.2 Blood collection, cell preparation, immunostaining and flow cytometry

Peripheral blood samples, 12ml in EDTA, were obtained from patients and healthy donors for comparative analysis. In patients, sampling was performed prior to the initiation of first-line treatment (cycle 1, day 1; C1D1), as well as at C1D15, C3D15, C6D15, and upon documentation of disease progression (PD). After collection, all samples were processed immediately for immunophenotypic analysis using flow cytometry. After red blood cell lysis, using Red Blood Cell Lysing buffer (BD Biosciences; USA) staining was performed for 30 min on ice in dark according to manufacturer’s instructions for surface markers. For FOXP3 staining, the cells were fixed and permeabilized using FOXP3 Buffer (BD Biosciences) according to manufacturer’s instructions and stained with Alexa-488 conjugated anti-FOXP3 antibody for 1 h on ice in dark. After washing, cells were re-suspended in 0.5 ml flow buffer and multicolor analysis was performed using a BD LSR II Flow Cytometer (BD Biosciences). FACS data were analyzed with Kaluza Software 2.1. The following monoclonal antibodies were used for immunophenotyping of Tregs: PE-Texas-Red conjugated anti-CD3, AmCyan-conjugated anti-CD4, PE-Cy7-conjugated anti-CD25, Alexa-488-conjugated anti-FOXP3 (Forkhead Box P3) and for MDSCs: anti-CD14 PE Cy7; Pacific Blue conjugated anti-CD15; FITC-conjugated-anti-CD11b; Alexa-700-conjugated-anti-CD33; APC-Cy7-conjugated-anti-HLA-DR; PE-conjugated-anti-Lin (CD3/CD4/CD16/CD56/CD19). All antibodies were obtained from BD Biosciences (USA). Staining for MDSCs and Tregs was performed in different tubes. The phenotypic marker used for identification of Tregs was CD3^+^CD4^+^CD25^+^FOXP3^+^, for polymorphonuclear MDSCs CD11b^+^CD14^-^CD15^+^CD33^+^HLA-DR^-^ and for monocytic MDSCs CD11b^+^CD14^+^CD33^+^CD15^-^HLA-DR^-^Lin^dim/-^. Detailed gating strategy is provided in the Supplementary Material.

Throughout this study, granulocytic subsets were operationally classified as “mature” (CD15^+^CD33^-^) and “immature” (CD15^+^CD33^+^), respectively. This classification is based on the known association of CD33 expression with earlier stages of myeloid differentiation; however, CD33 expression alone is not sufficient to definitively define neutrophil maturation stage. Therefore, these populations should be interpreted as phenotypically defined granulocyte subsets potentially enriched for different stages of maturation, rather than as discrete maturation stages. Accordingly, based on the expression of the CD16 neutrophil maturity marker, pMDSCs were further subdivided into a CD16^+^ mature (pMDSCs-mat) and CD16^dim/-^ immature subset (pMDSCs-im).

Frequencies of circulating Tregs (cTregs) were expressed as the proportion of FOXP3^+^ cells within the CD4^+^CD25^+^ population. Circulating monocytic myeloid-derived suppressor cells (cmMDSCs) were quantified as a fraction within monocytes (CD11b^+^CD14^+^ cells), while granulocytic subsets were assessed as frequencies within granulocytes (CD11b^+^CD14^-^ cells) or within their parent population based on CD15 and CD33 expression. For example, polymorphonuclear MDSCs (cpMDSCs) were also quantified as a fraction of CD15^+^CD33^+^ cells.

### 2.3 Statistical analysis

Given the exploratory nature of the present study, all statistical analyses were considered hypothesis-generating. Continuous variables were summarized as median and interquartile range (IQR), whereas categorical variables were summarized as frequencies and percentages.

Differences in immune cell frequencies between healthy donors and patients, as well as among different subgroups, were evaluated using the Wilcoxon–Mann–Whitney U test and are presented as boxplots displaying medians and IQRs. Correlations between immune cell populations were assessed using Spearman’s rank correlation coefficient, whereas Pearson’s correlation coefficient was used to evaluate linear associations between continuous variables where appropriate.

Longitudinal changes in immune cell subsets during treatment were analyzed using linear mixed-effects models, with sampling time point included as a fixed effect and patient as a random effect. To evaluate both early and late treatment-related changes, two complementary analyses were performed: C1D1 was used as the reference time point for comparisons with C1D15 and C3D15 whereas C3D15 served as the reference for comparisons with C6D15 and samples obtained at disease progression (PD). Analyses were performed for the entire cohort and separately within predefined clinical subgroups according to treatment response (CR/PR/SD versus PD), chemotherapy regimen (FOLFOX versus FOLFIRI), and targeted therapy (anti-VEGF versus anti-EGFR).

Progression-free survival (PFS) was defined as the time from treatment initiation to the first documented disease progression or death from any cause, whichever occurred first. Overall survival (OS) was defined as the time from treatment initiation to death from any cause. Patients without an event were censored at the date of the last follow-up. Median follow-up was estimated using the reverse Kaplan–Meier method. Hazard ratios (HRs) and 95% confidence intervals (CIs) were estimated using Cox proportional hazards regression. For immune cell subsets without previously established cut-off values, exploratory survival analyses were performed between the upper and lower halves of the cohort, the upper quartile versus the remaining patients, and the lower quartile versus the remaining patients.

Variables demonstrating a statistically significant association with survival in univariable Cox regression were subsequently entered into a multivariable Cox proportional hazards model. Statistical analyses and graphical representations were performed using R (35). All statistical tests were two-sided, and *P* < 0.05 was considered statistically significant.

Predefined cut-off values were adopted from previously published studies. Neutrophil counts ≥5,600/μL (24), monocyte counts ≥800/μL (36), and NLR values ≥3 (37) were considered elevated, whereas lymphocyte counts <1,000/μL were classified as low (7).

## 3 Results

Patients’ clinical characteristics are summarized in **Table 1** and the treatment characteristics of the cohort in **Supplementary Table 1**. With a median follow up of 22.7 months the median PFS of the cohort was 9 months and median overall survival 22.7 months. At baseline, 33 (37%) of the patients had neutrophilia, 17 (19%) had lymphopenia and 20 (22%) had lymphocytes higher than 2000/μl.

**Table 1:** Patients’ Characteristics. Absolute numbers are shown and percentages within brackets. CNS central nervous system.

Parameter		n (%)
	Median age	68
Gender	male	58 (65)
	female	32 (35)
ECOG PS	0/1	65 (72)
	2	25 (28)
Stage at Diagnosis	I-III	26 (28)
	IV	55 (61)
	NA	9 (10)
RAS status	mutant	39 (43)
	wild type	45 (50)
	NA	6 (7)
Sidedness	Left	79 ( 88)
	Right	11 (12)
Prior Adjuvant Chemotherapy	No	57 ( 63)
	Yes	24 (27)
	NA	9 (10%)
Metastatic sites	Liver	57 (63)
	Lung	33 (37)
	Lymphadenopathy	30 (33)
	Peritoneal	15 (17)
	Locoregional	28 (31)
	Bone	9 (10)
	Cutaneous	2 (2)
	CNS	2 (2)

### 3.1 Circulating immune cells before chemotherapy initiation

Initially, circulating immune cells with a potentially immunosuppressive function (cTregs and cMDSCs) were analyzed in both healthy donors (HD) and patients prior to the initiation of chemotherapy (C1D1). The proportion of cTregs within the CD4^+^CD25^+^ lymphocytic population was numerically lower in patients (median=13%, IQR = 20%) compared to HD (median=25%, IQR = 27%) but did not reach statistical significance (p=0.25). Similarly, cmMDSCs (CD11b^+^CD14^+^CD15^-^CD33^+^CD16^dim/-^HLADR^-^) as fraction of monocytic cells (CD11b^+^CD14^+^ myeloid cells) were numerically increased in patients (median=7.2%, IQR = 16.5%) compared to HD (median=2.9%, IQR = 6.3%, p=0.07) failing to reach statistical significance.

Circulating cells with a conventional polymorphonuclear MDSC phenotype (cpMDSCs-conv: CD11b^+^CD14^-^CD15^+^CD33^+^HLA-DR^-^) when estimated as fraction of polymorphonuclear cells (CD11b^+^CD14^-^) were significantly reduced in patients (median 9%, IQR 13%) relative to HD (median 20% IQR 13%, p=0.001). This population of cpMDSCs-conv was primarily composed of mature cells expressing CD16 (CD15^+^CD33^+^HLADR^-^CD16^+^, designated cpMDSCs-mat) and a minor fraction which was lacking or expressing low levels of CD16 indicative of an immature phenotype (CD15^+^CD33^+^HLADR^-^CD16^dim/-^, cpMDSCs-im). The former fraction, cpMDSCs-mat, was reduced in patients compared to healthy donors while the latter subset was significantly elevated (median=0.55‰ (IQR 1.49‰) compared to HD (median=0.22‰ (IQR = 0.34‰; p<0.001) (**Table 2**).

**Table 2:** Comparison of immune cell subsets between healthy donors and patients with metastatic colorectal cancer prior to chemotherapy initiation. Data are presented as median (IQR). Only immune cell subsets demonstrating statistically significant differences according to the Mann–Whitney U test are shown.

CD11b+CD14- subset	Healthy donors % (IQR)	Patients % (IQR)	p-values	Denominator
CD15+CD33-	73(15)	79(14)	0.003	CD11b+CD14-
CD15+CD33-HLADR-CD16+	69(16)	74(15)	0.03	
CD15+CD33-HLADR-CD16dim/-	0.8(1.1)	1.4(2.4)	0.026	
CD15+CD33-HLADR+CD16+	0.97(0.5)	0.44(0.9)	0.01	
CD15+CD33-HLADR+CD16+	1.5(0.7)	0.56(1.4)	0.008	CD15+CD33-
CD15+CD33+	21(12)	10(14)	0.002	CD11b+CD14-
CD15+CD33+HLADR-CD16dim/- (cpMDSCs-im)	0.02(0.03)	0.05(0.12)	0.01	
CD15+CD33+HLADR-CD16dim/- (cpMDSCs-im)	0.1(0.1)	0.55(1.7)	0.00002	CD15+CD33+
CD15+CD33+HLADR-CD16+ (cpMDSCs-mat)	21(12)	9(15)	0.002	CD11b+CD14-
CD15+CD33+HLADR+CD16+	0.4(0.45)	0.1(0.41)	0.006	
CD15-CD33-HLADR-CD16+	2.7(3.3)	0.8(2.1)	0.023	CD11b+CD14-
CD15-CD33-HLADR+CD16+	0.3(0.27)	0.03(0.12)	0.003	
CD15-CD33-HLADR+CD16+	9(7)	1.5(3)	0.0006	CD15-CD33-

**Figure 1:**
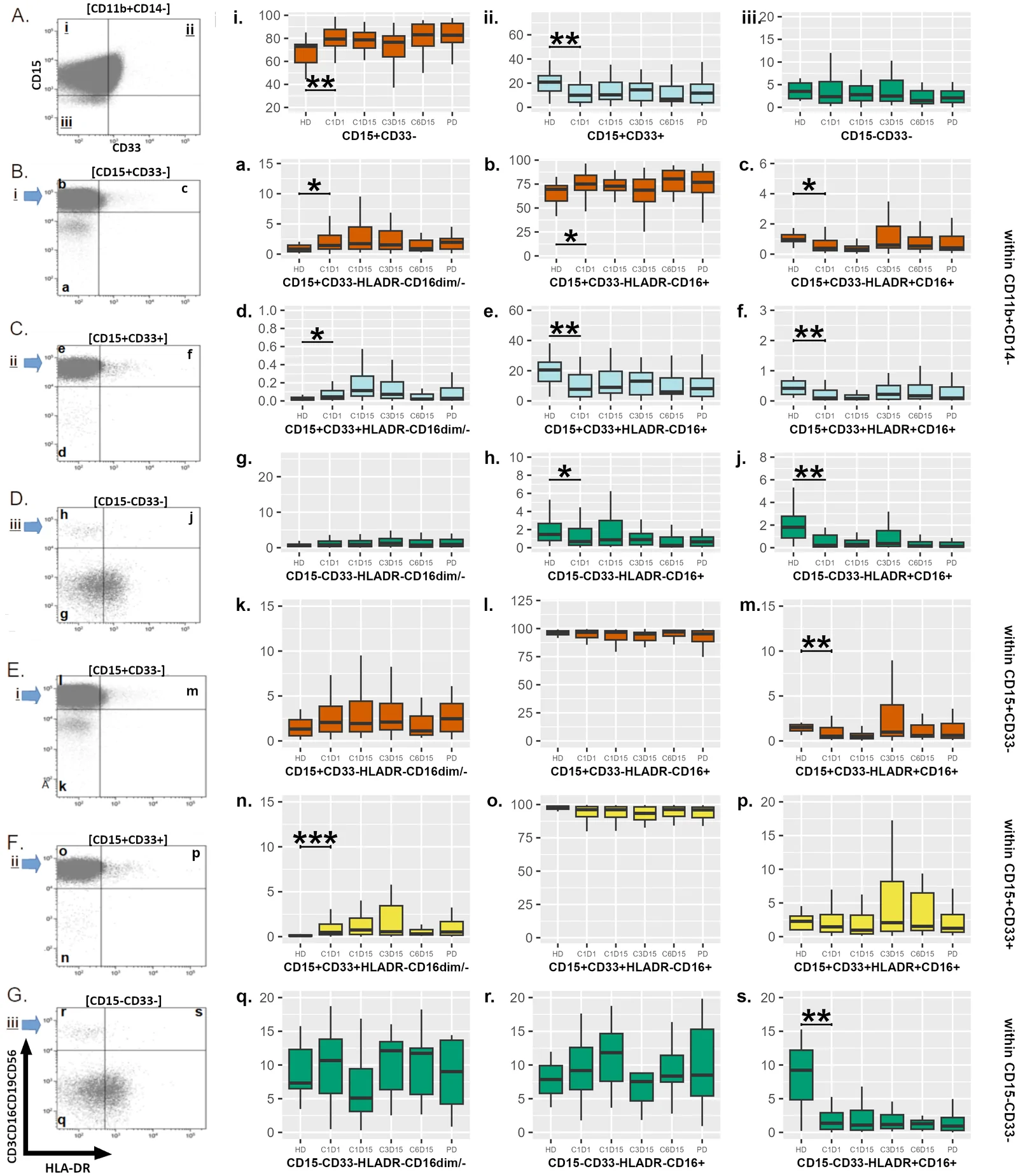
Quantification of granulocytic immune cell subsets by flow cytometry during chemotherapy. Each box in the box plots corresponds to either healthy donor (HD) samples or a specific sampling time point during chemotherapy for patients. CxDy Cycle x Day y of sampling. **(A–G)** Representative flow cytometry plots illustrating the gating strategy used to identify the corresponding granulocytic cell populations **(i–iii and a–s)**. **(i.–iii.)** Major granulocytic cell populations defined according to CD15 and CD33 expression and presented as the percentage of CD11b^+^CD14^-^ cells. **(a.–j.)** Granulocytic immune cell subsets defined by HLA-DR and CD16 expression shown as percentage of CD11b^+^CD14^-^ cells. **(k.–s.)** Granulocytic immune cell subsets expressed as percentage of their respective parental populations: **(k.–m.)** CD15^+^CD33^-^ cells, **(n.–p.)** CD15^+^CD33^+^ cells, and **(q.–s.)** CD15^-^CD33^-^ cells. Boxes indicate median values with interquartile range. Statistical significance was assessed using the Wilcoxon–Mann–Whitney U test (* for p < 0.05, ** for p < 0.01, *** for p < 0.001).

In addition to the aforementioned immune cell subsets, other granulocytic subpopulations identified from the flow cytometry plots were also compared between HD and patients. These populations were calculated either as fractions of CD11b^+^CD14^-^ cells (**Figures 1A–D, i–iii, a–j**) or as fractions of their immediate parent populations, namely within the CD15^+^CD33^+^, CD15^+^CD33^-^, or CD15^-^CD33^-^ compartments (**Figures 1E–G, k–s**). Several significant differences between healthy donors and patients were identified; they are presented in **Figure 1** and summarized in **Table 2**.

Overall, as shown in **Table 2**, mature granulocytes (CD15^+^CD33^-^) and their subsets were increased in patients compared to HD, whereas immature granulocytes (CD15^+^CD33^+^) and their subsets were generally decreased in patients. The only exception was the cpMDSCs-im subset, which was increased in patients due to a marked expansion within its parent population. This increase corresponded to median values approximately five-fold higher than those observed in HD and was associated with the lowest p-value in the comparison between healthy donors and patients. Lastly, the HLADR^+^CD16^+^ subsets of all three populations of myeloid cells were significantly reduced in patients both as fraction of total granulocytes and as fraction of their parent populations.

### 3.2 Correlation of immune cell populations with clinical parameters

Immune cell populations as quantified before chemotherapy initiation were compared within key clinical subgroups: presence of liver metastases, Eastern Cooperative Oncology Group (ECOG) performance status, sidedness of primary site (right versus left), presence of KRAS/NRAS/BRAF mutations or not in the cancer tissue, presence of neutrophilia or high NLR in the peripheral blood, response to treatment (CR/PR/SD versus PD) and stage of disease at first diagnosis (stage 1–3 versus 4). The significant results are shown in **Table 3**. In particular, patients with elevated absolute neutrophil counts (ANC) exhibited significantly reduced levels of cTregs in comparison to patients with lower neutrophil counts. This finding was similar when cTregs were calculated either as fraction of total lymphocytes or CD4^+^CD25^+^ lymphocytes. Furthermore, the absolute neutrophil count correlated inversely with cTregs (Spearmann’s Rho=-0.266, p=0.045) and the changes of neutrophils during chemotherapy also correlated inversely with the changes of cTregs (Spearmann’s Rho = -0.28, p=0.049).

**Table 3:** Differential distribution of immune cell subsets across clinical subgroups.

Subset	Median (IQR)		p-value	Denominator
Liver metastasis				
	Absent	Present		
Neutrophils	4500 (1500)	5500 (2300)	0.01	μl
CD15+CD33-	75% (14)	83% (11)	0.01	CD11b+CD14-
CD15+CD33+	15% (14)	6% (11)	0.007	
CD15+CD33+HLA-DR-CD16+	13% (14)	7% (12)	0.005	
CD15-CD33-HLA-DR-CD16dim-	0.9% (2)	0.7% (1)	0.044	
CD15+CD33+HLA-DR-CD16+	97% (3)	95% (18)	0.039	CD15+CD33+
CD15+CD33+HLA-DR+CD16+	1% (2)	2% (5)	0.029	
Location of primary				
	Left	Right		
CD15+CD33+HLA-DR+CD16+	0.15%(0.4)	0.03%(0.02)	0.008	CD11b+CD14-
CD15+CD33+HLA-DR+CD16+	1.9%(4)	0.5%(2)	0.03	CD15+CD33+
Stage at diagnosis				
	I-III	IV		
CD15+CD33+	17%(7)	15%(9)	0.013	CD11b+CD14-
CD15+CD33+HLA-DR-CD16+	14.8%(15)	6%(11)	0.018	
CD15-CD33-HLA-DR-CD16+	34%(45)	52%(40)	0.049	CD15-CD33-
Lymphocytes	1200(775)	1400(1000)	0.03	μl
Neutrophils	4450(2125)	5500(2150)	0.003	μl
Neutrophils				
	High	Low		
CD15+CD33-	86%(17)	78%(11)	0.01	CD11b+CD14-
CD15+CD33- HLA-DR-CD16+	83%(16)	73%(13)	0.037	
Tregs	6.7%(13)	17%(21)	0.043	CD4+CD25+

In patients with metastatic liver disease the neutrophil counts were significantly higher and the mature forms (CD15^+^CD33^-^) predominated while the immature neutrophils (CD15^+^CD33^+^) were reduced. A trend towards reduced cTreg levels (9.7% vs 16%, p=0.07) was also observed in patients with liver disease.

In patients who had been initially diagnosed with non-metastatic disease before developing metastatic disease, both absolute neutrophil and lymphocyte counts were reduced, whereas the proportion of immature neutrophils was increased. Additionally, the HLA-DR^+^CD16^+^ subset within the CD15^+^CD33^+^ granulocytic population was significantly lower in patients with right-sided primary tumors.

### 3.3 Correlations among immune cell populations

**Figure 2:**
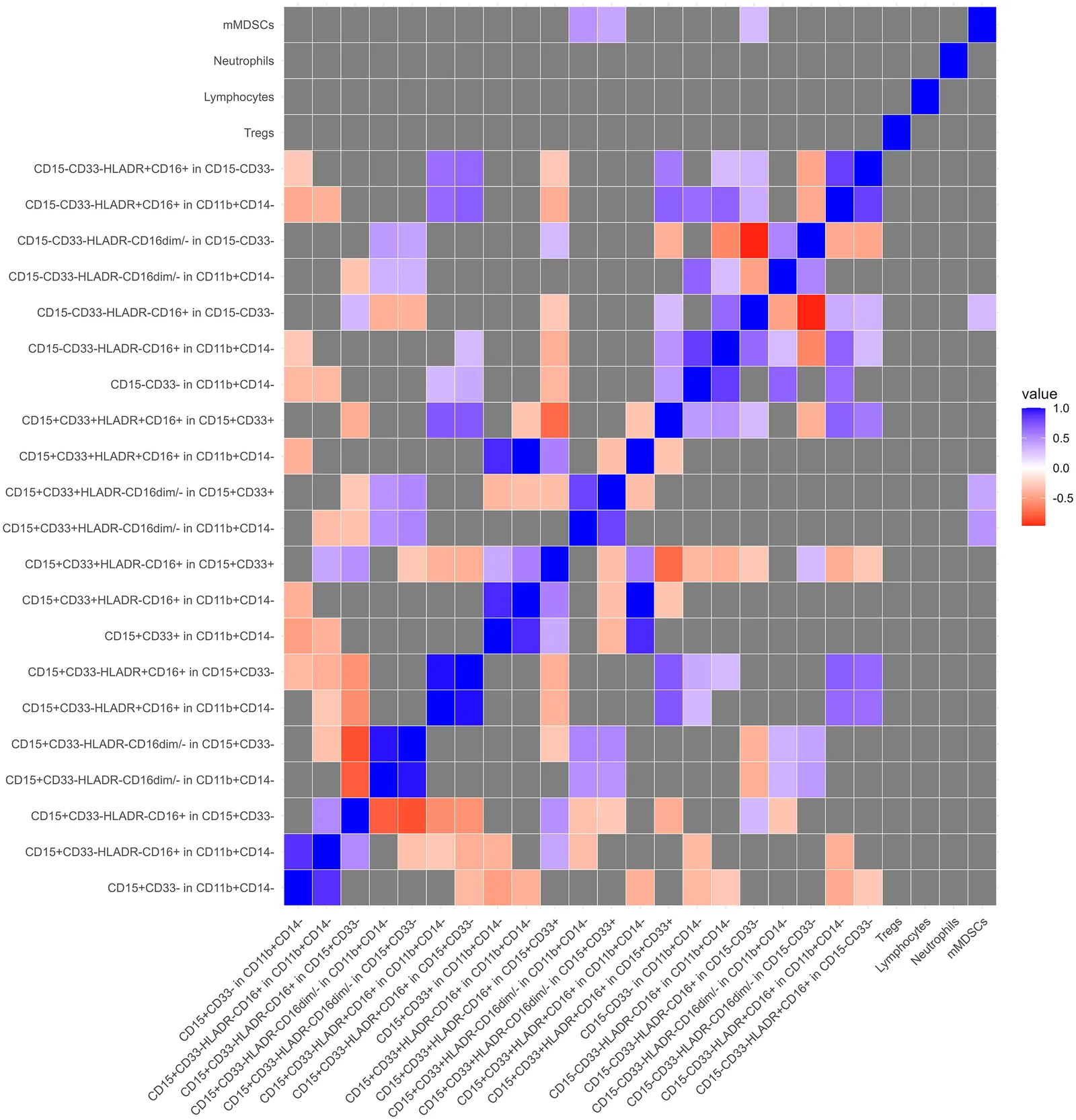
Heatmap of correlations between immune cell populations. Spearman’s correlation analysis among immune cell subsets is shown in form of a heat map. P-values for Spearmann’s Correlation were corrected for multiple comparisons and only significant correlations are shown. The rest is depicted in grey. As anticipated, populations with a common denominator in their quantification are correlated. Of note, also subsets with common patterns for CD16 and HLA-DR expression from different parent populations (eg. CD15^+^CD33^-^, CD15^+^CD33^+^) are also correlated with each other. The correlation between Tregs and neutrophils, although initially significant (*p* = 0.04), was no longer significant after correction for multiple comparisons. mMDSCs show a positive correlation with cpMDSCs-im.

Correlations among immune cell populations were explored using Spearman’s correlation coefficient, with p-values adjusted for multiple comparisons. A heat map summarizing significant correlations is presented in **Figure 2**. As anticipated, numerous associations were identified among immune cell subsets indicating a highly interconnected network. Several of these correlations were expected, reflecting relationships between phenotypic fractions within the same parent population. Accordingly, each parent population showed strong correlations with its most abundant phenotypic subset; for example, the CD15^+^CD33^-^ compartment correlated with its predominant HLA-DR^-^CD16^+^ fraction. Notably, correlations were also observed between subsets sharing similar patterns of HLA-DR and CD16 expression across different parent populations. For instance, HLA-DR^-^CD16^dim/−^ cells within the CD15^+^CD33^+^ compartment (corresponding to cpMDSCs-im) correlated with phenotypically analogous subsets within the CD15^+^CD33^-^ population, but not with those derived from the CD15^-^CD33^-^ compartment. This observation was also evident for HLA-DR^-^CD16^+^ and HLA-DR^+^CD16^+^ subsets which demonstrated consistent correlations across all three parent populations. Furthermore, monocytic MDSCs (CD11b^+^CD14^+^CD15^-^CD33^+^HLA-DR^-^, cmMDSCs) showed a more restricted pattern of association, correlating significantly only with cpMDSCs-im (CD15^+^CD33^+^HLA-DR^-^CD16^dim/−^) and CD15^-^CD33^-^HLA-DR^-^CD16^+^ cells. Finally, the inverse correlation between circulating Tregs and neutrophil counts did not retain statistical significance after correction for multiple testing, despite the observation that Treg levels were lower in patients with higher neutrophil counts.

Taken together, these findings suggest that myeloid populations defined by similar CD16 and HLA-DR expression patterns may be regulated in a coordinated manner across phenotypic compartments, potentially reflecting shared biological programs rather than isolated independent immune cell populations based on parent population assignment. These findings highlight potential limitations in evaluating the prognostic value of isolated immune subsets, underscoring the importance of considering their effect within a broader network.

To address the complexity of these interrelated immune cell populations and to explore whether immune profiles could discriminate between patients and healthy donors, K-means clustering was applied to the relative frequencies of myeloid populations. Given this objective, models with two or three centroids were initially considered. With two centroids, the vast majority of patients and healthy donors were assigned to the same cluster, indicating no meaningful separation between groups. With three centroids, one cluster contained the majority of both patients and healthy donors, while cluster 2 included a subset of patients along with only a limited number of controls. A third cluster comprised only two patients and likely represents outliers. Increasing the number of centroids did not result in improved separation of patients and healthy donors.

Comparison of cluster distribution between patients and healthy controls showed that Cluster 2 comprised 17.5% (14/80) of patient samples and 12.5% (2/16) of healthy donors; however, this difference did not reach statistical significance (Fisher’s exact test, *p* = 1). To further characterize the features distinguishing these clusters, a random forest classifier was applied, and feature importance was assessed using both Gini impurity and permutation-based accuracy measures. Both approaches consistently identified a set of variables as the primary drivers of cluster separation, with the highest-ranked features including the populations: CD15^-^CD33^-^HLA-DR^+^CD16^+^,CD15^+^CD33^-^HLA-DR^+^CD16^+^, CD15^+^CD33^+^HLADR^+^CD16^+^ and CD15^+^CD33^-^ in CD11b+CD14^-^. It is notable that mainly HLA-DR^+^CD16^+^ immune cell subsets drive cluster separation together with the subset of mature neutrophils.

These findings suggest that clustering based solely on relative myeloid cell frequencies may not be sufficient to discriminate between patients and healthy individuals. Rather, the observed clustering patterns may reflect distinct states of the myeloid cell compartment present in both healthy individuals and patients.

### 3.4 Immune cell dynamics during chemotherapy

We hypothesized that clinically or biologically relevant immune cell subsets will demonstrate significant changes during chemotherapy, reversing the differences observed between patients and healthy donors and restoring these changes towards baseline levels at disease progression; additionally these changes should be mainly present in responders while progressing patients should demonstrate an opposite deflection. Additionally, we wanted to explore whether different chemotherapeutic backbones or targeted agents had different effects on the kinetics of immune cell subsets; however a strict test for interaction of these parameters in the mixed effects model could not be performed because of insufficient sample size.

The graphs of immune cell populations during chemotherapy (**Figure 1**) indicated that chemotherapy-induced changes peaked at C3D15 followed by a reversal in trend. These observations were further investigated by longitudinal analyses, the results of which are summarized in **Supplementary Table 2** for statistically significant immune cell subsets, while the complete results for the overall cohort and predefined clinical subgroups are presented in **Supplementary Table 3**.

When analyzing population kinetics in responders (CR/PR/SD), there were two cell populations which demonstrated significant changes up to C3D15, which subsequently reversed and also demonstrated opposing trends in non-responders. These included mature neutrophils (CD15^+^CD33^-^), expressed as a fraction of the total granulocytic population, and their CD16-expressing subset. Notably, these changes tended to normalize the baseline differences observed between patients and healthy donors.

Other populations exhibited less pronounced alterations, reaching statistical significance only up to C3D15, with no significant changes thereafter, despite trends in the opposite direction. These included the HLA-DR^+^CD16^+^ subset of mature neutrophils, as well as the CD15^-^CD33^-^ populations in responders.

The immunosuppressive populations, including cpMDSCs-im and Tregs, showed a significant increase in responders, with opposite trends observed in non-responders. This increase in Tregs at C3D15 was particularly pronounced in patients achieving complete or partial response (CR/PR) (estimate at C3D15: +16%, *p* = 0.003). In contrast, circulating monocytic MDSCs (cmMDSCs) decreased at C3D15 in both responders and non-responders; however, this reduction reached statistical significance only in responders.

When juxtaposing different chemotherapy regimens, more pronounced immune cell changes were observed in patients receiving FOLFIRI (**Supplementary Table 3**) than in those who received FOLFOX, who, nevertheless, demonstrated similar directional trends. Of note, regulatory T cells showed significant increase only in the FOLFIRI cohort normalizing the baseline alteration observed in patients. Even more, analysis by targeted therapy revealed significant effects at all sampling time points of anti-EGFR treatment on the cpMDSCs-mat population characterized by an increase up to C3D15 followed by a decline thereafter, a pattern that was not observed in any other subset. In contrast, antiangiogenic therapy was associated with an opposite pattern that did not reach statistical significance in this population (**Supplementary Table 3**).

### 3.5 Prognostic significance of circulating immune cells

In our cohort several clinical parameters showed correlation with overall survival (OS) but not with progression free survival (PFS) in accordance with established prognostic factors (i.e. stage at diagnosis; liver metastasis; prior adjuvant therapy (**Supplementary Table 4**).

From the common blood based markers (**Supplementary Table 5**) a high neutrophil count and -to a lesser extent- a high NLR also correlated with worse survival. Circulating lymphocytes demonstrated significance in cox-regression as a continuous variable, comparable to neutrophils in effect size, correlating with worse outcome. By stepwise testing, a cut-off of 1900 lymphocytes/μl was detected to provide a significant difference in OS by log-rank test (p=0.03).

Among immune cell populations with an immunosuppressive phenotype, higher frequencies of cmMDSCs were associated with inferior OS and PFS; cpMDSCs-im demonstrated a trend towards worse OS that did not reach statistical significance while cpMSCS-mat correlated with favorable PFS but not OS. Regarding cTregs there was no prognostic significance neither for OS nor for PFS.

Among exploratory myeloid subpopulations, patients with a lower mature neutrophil fraction (CD15^+^CD33^-^) within the CD11b^+^CD14^-^ compartment demonstrated better OS. The subsets of mature neutrophils according to the expression of CD16 demonstrated opposite prognostic behavior in terms of PFS. Specifically the CD16+ fraction correlated with favorable prognosis while the CD16dim/- fraction demonstrated an opposite effect with dismal prognosis at different cut-offs and denominators.

Variables demonstrating a statistically significant association with overall survival in the univariable Cox regression analysis were subsequently entered into a multivariable Cox proportional hazards model. The model included absolute neutrophil count, absolute lymphocyte count, mature neutrophils, mMDSCs, ECOG performance status, liver involvement, and stage at diagnosis. In the multivariable analysis, ANC (HR 1.00015, 95% CI 1.000017–1.000285, *P* = 0.03) and circulating mMDSCs (HR 2.06, 95% CI 1.03–4.14, *P* = 0.04) remained independently associated with overall survival.

## 4 Discussion

In the current study, we characterized circulating immune cell populations in a cohort of metastatic colorectal cancer patients undergoing first-line chemotherapy and examined their associations with clinical parameters, and outcome. Our findings highlight substantial heterogeneity within circulating immune cell compartments and challenge prevailing assumptions regarding the role of specific cell populations.

A key observation of the present study is the inverse association between circulating CD4^+^CD25^+^FOXP3^+^ regulatory T cells (cTregs) and neutrophil counts, which was evident both in pretreatment baseline samples and in the longitudinal changes of these cell populations during chemotherapy. Elevated cTreg levels—comparable to those of healthy donors—were observed primarily in patients with low neutrophil counts, whereas neutrophilia was associated with a contraction of the cTreg compartment. To the best of our knowledge, this association has not been described before and represents a novel finding. In contrast to the commonly held view that circulating Tregs are expanded in cancer (8, 38, 39), Treg levels in our cohort were not increased compared to healthy donors and were, in fact, numerically reduced, particularly in patients with neutrophilia. Reduced Treg frequencies have been previously reported in colorectal cancer patients (26), while other studies have described increased (25, 40, 41) or unchanged levels (42). Importantly, most of these studies included predominantly non-metastatic or heterogeneous patient populations, limiting direct comparisons.

The present study addresses the need for investigating cTregs in a well-defined cohort of metastatic colorectal cancer patients initiating first line chemotherapy. Our observations suggest that differences in neutrophil counts across different studies may partly explain the conflicting reports regarding circulating Treg frequencies in CRC.

The inverse association between cTregs and neutrophils may reflect interactions between the adaptive and innate immune compartments in advanced disease. An additional explanation could be that cTregs, rather than representing a stable immunosuppressive population directly driving tumor progression, may be regulated by the systemic inflammatory milieu shaped by the tumor, stromal cells and potentially coexisting microbiota: cytokines, growth factors and chemokines that promote neutrophil expansion may exert opposing effects on Tregs or induce their homing to peripheral compartments. Consistent with both of these interpretations cTreg levels were not prognostic in the present cohort.

An alternative explanation for this inverse association between cTregs and ANC could be the redistribution of Tregs between the peripheral blood and the cancer tissue or peripheral lymphoid organs leading to their depletion in the periphery. In further support of this notion, cTregs increased significantly in responders but decreased further in progressors, unraveling an indirect inverse correlation with disease burden. This supposition is consistent with previous observations in ovarian cancer where Tregs were enriched within the tumor tissue while being depleted in draining lymph nodes (18) suggesting the existence of a limited pool of Tregs rather than an unlimited reserve.

Although technical variability inherent to multiparametric flow cytometry cannot be completely excluded, the standardized gating strategy applied throughout the study and the reproducibility of the inverse association in both pretreatment and longitudinal samples make a purely technical explanation unlikely. However, given the observational nature of this analysis these assumptions remain hypothesis generating. The inverse correlation between cTregs and ANC should first be validated in independent cohorts and the underlying mechanisms elucidated in studies integrating immune profiling with histological findings.

Another notable finding from this study pertains to the absolute lymphocyte count (ALC). Lymphocytopenia has been consistently linked to worse prognosis in cancer which was also confirmed in multiple studies in non-metastatic colorectal cancer (43–46). In metastatic CRC, on the other hand, the lymphocytes are usually integrated within a composite marker like the NLR (47), the lymphocyte to monocyte ratio (LMR) (48), the platelet-lymphocyte ratio (PLR) (49), the prognostic nutritional index (PNI) (50), albumin-total lymphocyte count-RAS index (ALRI) (51). The prognostic significance of the ALC alone in mCRC has received little attention.

In the present cohort, ALC exhibited considerable interindividual variability, with patients presenting with reduced, normal, or increased lymphocyte counts. Importantly, ALC was significantly associated with overall survival both when analyzed as a continuous variable and when dichotomized using a cut-off value of 1,900 cells/μL. These findings suggest that ALC may have potential utility as a prognostic biomarker in metastatic disease.

An unexpected observation was the significantly higher ALC in patients presenting with de novo metastatic disease despite their inferior survival compared to patients who developed metastatic disease following treatment for initially localized cancer. One possible explanation is that patients with metachronous metastatic disease had previously been exposed to surgery, chemotherapy, and/or radiotherapy, all of which may affect peripheral lymphocyte counts. Alternatively, prolonged interaction between the immune system and the primary tumor in patients with metachronous metastatic disease before metastatic progression may have resulted in immune editing, thereby altering the host immune response once metastatic disease developed. This finding indicates that the biological significance of peripheral lymphocyte counts may differ according to disease presentation and treatment history. Although these hypotheses remain speculative, they merit prospective investigation.

Our findings also highlight a potential limitation of composite inflammatory indices that incorporate lymphocyte count like the NLR. By expressing lymphocyte count as a ratio with other leukocyte populations, these indices may mask biologically meaningful heterogeneity. For example, patients with similar NLR values may have markedly different absolute neutrophil and lymphocyte counts, potentially reflecting distinct inflammatory and immunological states. Therefore, while combined indices such as NLR remain clinically valuable prognostic biomarkers, the present results suggest that absolute lymphocyte count may provide complementary prognostic information that is not fully captured by composite ratios. Validation of these observations in independent prospective cohorts is warranted to determine whether ALC should be considered as an additional prognostic biomarker in metastatic colorectal cancer.

Another observation from our study is that CD15^+^CD33^-^ polymorphonuclear cells, (operationally defined as “mature neutrophils”), were increased in patients compared to healthy controls, with a more pronounced enrichment in patients with adverse clinical features such as liver involvement and neutrophilia. Longitudinally, the dynamics of this population differed according to response to treatment. In responding patients, mature neutrophils decreased during the initial cycles of chemotherapy and subsequently increased at later time points and at disease progression, whereas in non-responders a non-significantt increase was observed. Cautious interpretation is warranted as the sample size precluded formal statistical evaluation of the relationship between response to treatment and immune cell population kinetics. Additionally, lower levels of CD15^+^CD33^-^ polymorphonuclear cells were associated with improved overall survival. These patterns suggest that in patients with mCRC, the changes of frequency of CD15^+^CD33^-^ polymorphonuclear cells during treatment represent a dynamic marker for disease activity. Furthermore, these findings suggest that, in mCRC, expansion of granulocytic populations is not restricted to immature subsets but mainly involves phenotypically mature neutrophils, supporting a model of functional reprogramming rather than immature granulopoiesis (11, 52, 53).

The CD15^+^CD33^-^ compartment is composed predominantly of HLA-DR^-^CD16^+^ cells and two minor fractions HLA-DR^-^CD16^dim/−^ and the HLA-DR^+^CD16^+^. The HLA-DR^-^CD16^+^ fraction was enriched in patients, consistently with its parent population and was further increased in patients with neutrophilia. Moreover, it exhibited the same pattern of changes during treatment as the CD15^+^CD33^-^ parent population and was associated with more favorable PFS. In contrast, enrichment of the HLA-DR^-^CD16^dim/−^ subset correlated with worse PFS. Overall, these data further support enrichment of mature neutrophils in the peripheral blood of mCRC patients and suggest a distinct, potentially adverse role for the HLA-DR^-^CD16^dim/−^ subset.

Granulocytic cells with a phenotype consistent with pMDSCs (CD15^+^CD33^+^HLA-DR^-^, cpMDSCs-conv) were reduced in patients compared to healthy donors and the CD16^+^ fraction (cpMDSCs-mat) followed the same pattern. This observation contrasts with previous reports employing similar, though not identical, phenotypic definitions for pMDSCs (33, 34). In addition, patients with liver involvement exhibited reduced frequencies of cpMDSCs-mat. On the other hand, the CD16^dim/−^ subpopulation (cpMDSCs-im) followed an opposite pattern; it was selectively enriched in patients, reaching the highest level of statistical significance among all comparisons performed between HD and patients. Elevated levels of cpMDSCs-im were associated with a trend toward inferior overall OS, consistent with prior reports using similar phenotypic definitions, whereas higher levels of the CD16^+^ subset were associated with improved PFS. Taken together, these findings suggest that the conventional phenotypic definition of pMDSCs contains distinct CD16-defined subsets with CD16^+^ populations associated with favorable clinical parameters while CD16^dim/-^ subsets correlating with clinical features consistent with what is expected from immunosuppressive pMDSCs.

Regarding monocytic MDSCs, they were only modestly increased in patients but were nonetheless associated with adverse OS and PFS which further corroborates a previous report (33). This apparent discrepancy between limited quantitative differences and clear prognostic relevance suggests that functional or phenotypic heterogeneity within the cmMDSC compartment may not be adequately addressed by current marker definitions. Further studies are warranted to further refine the complexity of this population. Furthermore, cmMDSCs were correlated with cpMDSCs-im indicating potential coordinated regulation between the two subsets and suggesting that these populations should not be evaluated in isolation.

Finally HLA-DR+CD16+ subsets were consistently identified across all major granulocytic populations. HLA-DR is an antigen presenting molecule typically absent in neutrophils and HLA-DR+ cells are considered rare within the granulocytic compartment. Increased frequency has been reported under different inflammatory conditions (54). Additionally, human neutrophils are known to be able to differentiate into hybrids between neutrophils and dendritic cells (55) or monocytes (56) under specific conditions expressing HLA-DR and possessing antigen presenting properties. HLA-DR+ neutrophils have also been detected with single cell sequencing of tumor infiltrating neutrophils and have been associated in diverse cancer types with favorable prognosis indicating an anti-tumor action (57). They have not yet attracted attention as circulating biomarker in cancer patients. In line with the above findings in our cohort their frequency was indeed less than 1% in healthy donors. In patients they were significantly reduced compared to healthy donors and even more so in patients with right-sided primary while higher levels were found in patients with liver involvement. Furthermore, their kinetics during chemotherapy seemed to inversely follow tumor volume and activity suggesting either active suppression by tumor or increased recruitment and consummation from the periphery into tumor site.

Comparison of the effect of treatment regimens on population kinetics suggested differential effects of chemotherapy backbones on specific immune cell populations. Regulatory T cells changed significantly only in the FOLFIRI cohort, whereas patients receiving anti-EGFR therapy exhibited significant alterations across all sampling time points in the cpMDSCs-mat subset. These findings suggest treatment-specific modulation of circulating immune populations and warrant further investigation. Furthermore, the observation that multiple immune cell populations exhibit divergent changes in frequency during chemotherapy in responders versus progressors suggests that these alterations are not solely attributable to the cytotoxic effects of treatment. Rather, they likely reflect the dynamic influence of tumor burden on the systemic inflammatory milieu.

Several limitations should be acknowledged in our study. The study is observational and based primarily on relative cell frequencies, while absolute measurements may provide additional information on the studied immune cell populations. Due to limitations of the sample size longitudinal analyses were limited. Given the observational nature of this study and the lack of functional validation or validation in a separate cohort these results should be considered hypothesis-generating.

Taken together, these findings support a model in which distinct components of the systemic immune response contribute complementary prognostic information. Whereas the absolute neutrophil count and circulating monocytic MDSCs primarily reflect adverse myeloid-driven inflammation and immunosuppression, the lymphocyte count and phenotypically distinct mature neutrophil subsets appear to capture additional aspects of host response and disease progression. Furthermore, phenotypic characterization allowed further subdivision of major myeloid cell populations, including neutrophils and polymorphonuclear MDSCs, into distinct subsets with different distributions across clinical subgroups and divergent prognostic associations.

In view of the expanding therapeutic landscape in colorectal cancer, including immunotherapeutic and other immune-modulating approaches, future studies should determine whether comprehensive immune profiling can improve patient stratification, inform treatment selection and identify patients most likely to benefit from therapies directed against specific immune cell populations.

## Data Availability

The raw data supporting the conclusions of this article will be made available by the authors, without undue reservation.
