# Supplementary material for: Coordinated dynamics of circulating neutrophils, regulatory T lymphocytes, myeloid derived suppressor cells and distinct granulocytic subsets reveal a complex immune network in metastatic colorectal cancer
Source: Front Oncol. 2026 Aug 4;16:1873352. doi: 10.3389/fonc.2026.1873352 (PMC13508029; doi:10.3389/fonc.2026.1873352)
Supplement: Supplementary file 1 [file DataSheet1.docx]

Supplementary Material

# 1 Gating Strategy

For immunophenotyping more than 100,000 cells were acquired from each sample (median 10^6^, IQR 590,000-2,220,000). The gating strategy used is shown in Supplementary Fig.1. After excluding doublets the phenotypic marker used for identification of Tregs was CD3^+^CD4^+^CD25^+^FOXP3^+^ cells. The analysis was restricted in the lymphocytic population from the SSC-Α vs FSC-Α plot, from which sequentially CD3^+^ cells, CD4^+^, CD25^+^ and FOXP3^+^ lymphocytes were gated. The fluorescence value of PE-Cy7-conjugated anti-CD25 where less than 0.5% of the CD4^-^ lymphocytes had higher fluorescence values served as gating threshold for CD25 positivity for CD4^+^ cells in accordance with previous reports using CD4^-^ cells as internal negative control for CD25 gating (15). In a similar way, the fluorescence value of FOXP3 at which less than 0.5% of CD4^+^CD25^-^ lymphocytes were positive served as threshold for positivity for CD4^+^CD25^+^ cells (Supplementary Figure 1).

After excluding doublets myelocytes were initially identified based on forward and side scatter characteristics. Subsequently CD11b+ cells were further analyzed into CD14- which correspond to granulocytes and CD14+ corresponding to monocytes. For granulocytes subpopulations were defined according to CD33 and CD15 expression as CD15⁺CD33⁻, CD15⁺CD33⁺ and CD15^-^CD33^-^ cells. Monocytes were further analyzed according to CD15 and CD33 expression and CD15-CD33+ cells were selected to be analyzed with HLA-DR and CD16. For MDSCs characterization two phenotypic definitions were used: CD11b^+^CD14^-^CD15^+^CD33^+^HLADR^-^Lin^dim/-^ for polymorphonuclear MDSCs and CD11b^+^CD14^+^CD33^+^CD15^-^HLADR^-^Lin^dim/-^ for monocytic MDSCs. The anti-Lin antibody included anti-CD3, CD16, CD19, CD56 antibodies. Since the gating for MDSCs was performed only in cells of the myeloid lineage as per SSC-Α vs FSC-Α and lymphocytes were excluded, Lin^+^ cells practically represented CD16^+^ cells, the only marker of the Lin cocktail expressed in the myeloid lineage. Given the applied gating strategy, CD16 expression was approximated by Lin expression for the purposes of this study.

# 2 Supplementary Figures and Tables

## 2.1 Supplementary Figures

Supplementary Figure 1 Gating strategy for the identification of regulatory T lymphocytes (Tregs).Lymphocytes were initially identified based on forward scatter and side scatter characteristics. The lymphocyte population was subsequently analyzed for CD3 and CD4 expression, and CD4⁺ T cells were selected. Within this population, cells were gated for CD25 expression and the CD4⁺CD25⁺ subset was further analyzed for FOXP3 expression. Regulatory T cells were defined as FOXP3⁺ cells within the CD4⁺CD25⁺ population. To determine the threshold for CD25 positivity, CD4⁻ cells were used as internal negative controls, with the cutoff set at 0.5% while the threshold for FOXP3 positivity was established using CD4⁺CD25⁻ cells with the cutoff at 0.5%.

Supplementary Figure 2 Gating strategy for the identification of MDSC subsets by flow cytometry. Myeloid cells were first selected based on forward and side scatter characteristics. CD11b⁺ cells were subsequently analyzed for CD14 expression to distinguish monocytic (CD14^+^) and granulocytic (CD14^-^) populations. CD11b⁺CD14⁻ cells were further gated for CD15 and CD33. CD15^+^CD33^+^ cells were then subdivided according to HLA-DR and CD16 expression. These cells were then subdivided according to HLA-DR expression into CD16^dim/-^ immature (pMDSCs-im) and CD16^+^ mature (pMDSCs-mat) subsets. Both of them combined constitute conventional polymorphonuclear MDSCs (pMDSCs-conv). Monocytic MDSCs (mMDSCs) were defined as CD11b⁺CD14⁺CD15⁻CD33⁺HLA-DR⁻ cells. Negative controls were used to establish gating thresholds are shown on the left.

## 2.2 Supplementary Tables

Supplementary Table 1 Treatment characteristics of the study cohort, including chemotherapy regimen, targeted therapy and treatment response.

| Parameter | | n(%) |
| --- | --- | --- |
| Chemotherapy | 5-FU | 2 (2) |
|  | FOLFIRI | 60 (67) |
|  | FOLFOX | 26 (29) |
|  | FOLFOXIRI | 2 (2) |
| Targeted Agent | Antiangionesis | 40 (44) |
|  | Anti-EGFR | 31 (34) |
| Response | PD | 17 (19) |
|  | CR | 2 ( 2) |
|  | PR | 35 (39) |
|  | SD | 34(38) |
|  | NA | 2(2) |

Supplementary Table 2 Longitudinal changes in immune cell subsets assessed using linear mixed-effects models. C1D1 was used as the reference for comparisons with C1D15 and C3D15, whereas C3D15 was used as the reference for comparisons with C6D15 and disease progression (PD). Only subsets with statistically significant results are shown.

|  |  |  | **C1D15** | | **C3D15** | | **C6D15** | |  | **PD** | |
| --- | --- | --- | --- | --- | --- | --- | --- | --- | --- | --- | --- |
| **As fraction in** | **Population** | **Response** | **Estimate** | **p** | **Estimate** | **p** | **Estimate** | **p** |  | **Estimate** | **p** |
| **CD11b+CD14-** | **CD15+CD33-** | **CR/PR/SD** | -0.89 | 0.724 | **-5.95** | **0.021** | **8.75** | **0.021** |  | **8.26** | **0.02** |
|  |  | **PD** | 2.76 | 0.75 | 15.09 | 0.388 |  |  |  | -4.79 | 0.812 |
|  | **CD15+CD33- HLADR-CD16+** | **CR/PR/SD** | -3.18 | 0.284 | **-9.64** | **0.002** | **12.61** | **0.011** |  | 7.42 | 0.106 |
|  |  | **PD** | 5.72 | 0.554 | 17.77 | 0.373 |  |  |  | -4.82 | 0.817 |
|  | **CD15+CD33- HLADR+CD16+** | **CR/PR/SD** | 0.96 | 0.462 | **2.98** | **0.026** | -2.98 | 0.13 |  | -0.50 | 0.788 |
|  |  | **PD** | -2.65 | 0.333 | -2.60 | 0.629 |  |  |  | 0.04 | 0.931 |
|  |  |  |  |  |  |  |  |  |  |  |  |
|  |  |  |  |  |  |  |  |  |  |  |  |
|  | **CD15+CD33+** | **CR/PR/SD** | **4.34** | **0.029** | 3.04 | 0.128 | -2.12 | 0.49 |  | -0.93 | 0.74 |
|  |  | **PD** | 1.89 | 0.493 | **-14.18** | **0.048** |  |  |  | 1.22 | 0.714 |
|  | **CD15+CD33+HLADR-CD16^dim/-^** | **CR/PR/SD** | -0.05 | 0.679 | **0.28** | **0.034** | -0.08 | 0.753 |  | -0.28 | 0.251 |
|  |  | **PD** | -0.32 | 0.613 | -0.34 | 0.797 |  |  |  | 0.09 | 0.871 |
|  |  |  |  |  |  |  |  |  |  |  |  |
|  | **CD15-CD33-HLADR+CD16+** | **CR/PR/SD** | 0.05 | 0.703 | **0.29** | **0.022** | -0.37 | 0.252 |  | 0.19 | 0.525 |
|  |  | **PD** | -0.20 | 0.29 | -0.25 | 0.516 |  |  |  | 0.01 | 0.901 |
| **CD15+CD33-** | **CD15+CD33-HLADR-CD16+** | **CR/PR/SD** | -1.69 | 0.382 | **-4.82** | **0.014** | 4.17 | 0.246 |  | 0.21 | 0.95 |
|  |  | **PD** | 3.52 | 0.411 | 1.55 | 0.822 |  |  |  | 0.00 | 0.998 |
|  | **CD15+CD33-HLADR+CD16+** | **CR/PR/SD** | 0.85 | 0.609 | **3.82** | **0.023** | -4.34 | 0.103 |  | -0.14 | 0.956 |
|  |  | **PD** | -3.13 | 0.318 | -3.17 | 0.517 |  |  |  | 0.00 | 0.998 |
|  |  |  |  |  |  |  |  |  |  |  |  |
| **CD15-CD33-** | **CD15-CD33-HLADR-CD16+** | **CR/PR/SD** | **-8.44** | **0.041** | **-13.95** | **0.001** | 3.31 | 0.677 |  | -3.00 | 0.69 |
|  |  | **PD** | 6.53 | 0.628 | -9.25 | 0.67 |  |  |  | 0.00 | 1 |
|  | **CD15-CD33-HLADR+CD16+** | **CR/PR/SD** | 2.74 | 0.153 | **4.84** | **0.012** | -5.20 | 0.149 |  | 2.09 | 0.538 |
|  |  | **PD** | -4.06 | 0.109 | -4.47 | 0.256 |  |  |  | 0.00 | 0.998 |
|  |  |  |  |  |  |  |  |  |  | |  |
| **CD4+CD25+** | **Tregs** | **CR/PR/SD** | 5.53 | 0.16 | **8.85** | **0.032** | 4.73 | 0.461 | -7.03 | | 0.249 |
|  |  | **PD** | 11.37 | 0.077 | -14.19 | 0.087 |  |  |  | 2.94 | 0.835 |
| **CD11b+CD14+** | **mMDSCs** | **CR/PR/SD** | -1.80 | 0.28 | **-3.77** | **0.028** | -0.49 | 0.868 |  | 1.44 | 0.601 |
|  |  | **PD** | 8.70 | 0.257 | -3.13 | 0.798 |  |  |  | 0.00 | 0.997 |

**Supplementary Table 3**(attached as a separate file). Complete results of the longitudinal analyses of all immune cell populations for the overall cohort and the predefined clinical subgroups.

Supplementary Table 4 Clinical prognostic factors for overall survival (OS) and progression-free survival (PFS). Hazard ratios (HRs), 95% confidence intervals (CIs), and p-values are presented. Statistically significant results (p< 0.05) are shown in bold.

|  | **OS** | | | | **PFS** | | | |
| --- | --- | --- | --- | --- | --- | --- | --- | --- |
| **Parameter** | **HR** | **Conf. Interv. 95%** | | **p** | **HR** | **Conf. Interv. 95%** | | **p** |
| Age at stage IV ≥65 | 1.3 | 0.76 | 2.25 | 0.32 | 0.95 | 0.56 | 1.61 | 0.85 |
| Stage at Diagnosis IV vs I-III | 2.12 | 1.13 | 3.95 | **0.019** | 1.34 | 0.77 | 2.36 | 0.30 |
| Liver Metastasis Present Yes vs No | 1.84 | 1.04 | 3.24 | **0.035** | 1.16 | 0.68 | 1.99 | 0.58 |
| Location of primary Right vs Left | 1.79 | 0.87 | 3.67 | 0.11 | 1.17 | 0.59 | 2.32 | 0.65 |
| RAS family WT vs mutant | 0.74 | 0.44 | 1.25 | 0.265 | 0.9 | 0.5 | 1.6 | 0.8 |
| Response CR/PR/SD vs PD | 0.11 | 0.06 | 0.21 | **0.000** | NA | NA | NA | NA |
| Prior adjuvant yes vs no | 0.49 | 0.26 | 0.94 | **0.03** | 0.82 | 0.45 | 1.49 | 0.51 |

Supplementary Table 5 Prognostic significance of immune cell populations for overall survival (OS) and progression-free survival (PFS). Hazard ratios (HRs), 95% confidence intervals (CIs), and *P* values are presented. Statistically significant associations (*P* < 0.05) are shown in bold.

|  | | | **OS** | | | | **PFS** | | | | | | | |
| --- | --- | --- | --- | --- | --- | --- | --- | --- | --- | --- | --- | --- | --- | --- |
| **Parameter** | | | **HR** | **Conf. Interv. 95%** | | **p** | **HR** | **Conf. Interv. 95%** | | | | | **p** | |
| Neutrophils/μl (1/μλ) | | | 1.00019 | 1.000028 | 1.000346 | **0.021** | 1.00009 | 0.9999914 | | | 1.0002 | | 0.07 | |
| Neutrophils low (<5600/μl) vs high | | | 0.42 | 0.25 | 0.71 | **0.001** | 0.90 | 0.54 | | | 1.50 | | 0.70 | |
| Lymphocytes/μl | | | 1.00044 | 1.0001 | 1.00078 | **0.012** | 1.00033 | 0.9999848 | | | 1.0007 | | 0.06 | |
| Lymphocytes low (<1900/μl) vs high | | | 0.62 | 0.42 | 0.92 | **0.019** | 0.82 | 0.55 | | | 1.22 | | 0.33 | |
| NLR | | | 1.11 | 1.016 | 1.213 | **0.028** | 1.03 | 0.94 | | | 1.13 | | 0.49 | |
| NLR low (<3 ) vs high | | | 0.59 | 0.35 | 1.001 | 0.046 | 0.71 | 0.42 | | | 1.20 | | 0.20 | |
| cpMDSCs-im  >50% vs rest | | | 1.6 | 0.92 | 2.94 | 0.09 | 0.99 | 0.53 | | | 1.86 | | 0.98 | |
| cpMDSCs-mat (CD11b^+^CD14^-^CD15^+^CD33^+^HLADR-CD16^+^ ) in CD15^+^CD33^+^  (highest half vs lowest) | | | 0.86 | 0.49 | 1.53 | 0.62 | 0.56 | 0.31 | | | 0.99 | | **0.048** | |
| cmMDSCs  ≥75% vs rest | | | 1.98 | 1.04 | 3.78 | **0.039** | 2.20 | 1.2 | | | 4.3 | | **0.018** | |
| Tregs  ≥75% vs rest | | | 1.06 | 0.46 | 2.5 | 0.9 | 1.36 | 0.62 | | | 2.99 | | 0.44 | |
| CD11b^+^CD14^-^CD15^+^CD33^-^ within CD11b^+^CD14^-^  ( lowest quartile vs rest) | | | 0.54 | 0.3 | 0.98 | **0.045** | 0.8 | 0.5 | | | 1.4 | | 0.5 | |
| CD11b^+^CD14^-^ CD15^+^CD33^-^ HLADR^-^CD16^+^ in CD15^+^CD33^-^  (highest half vs rest) | | | 0.93 | 0.52 | 1.64 | 0.79 | 0.36 | 0.20 | | | 0.65 | | **0.001** | |
| CD11b^+^CD14^-^ CD15^+^CD33^-^ HLADR^-^CD16^dim/-^ in CD15^+^CD33^-^  (highest half vs rest) | | | 1.01 | 0.56 | 1.85 | 0.96 | 2.16 | 1.20 | | | 3.89 | | **0.01** | |
| CD11b^+^CD14^-^CD15^+^CD33^-^HLADR^-^CD16^dim/-^ in CD11b^+^CD14^-^  (highest half vs lowest) | | | 0.98 | 0.55 | 1.74 | 0.95 | 2.44 | 1.32 | | | 4.52 | | **0.005** | |
| CD15^+^CD33^-^HLADR^-^CD16^dim/-^ in CD11b^+^CD14^-^  (highest quartile vs rest) | | | 1.29 | 0.66 | 2.53 | 0.45 | 2.0 | 1.1 | | | 3.8 | | **0.03** | |
| CD15^-^CD33^-^ / CD11b^+^CD14^-^  (highest quartile vs rest) | | | 1.16 | 0.58 | 2.31 | 0.68 | 2.2 | 1.1 | | | 4.2 | | **0.02** | |
| CD15^-^CD33^-^HLADR^-^CD16^dim/-^ in CD11b^+^CD14^-^  (highest quartile vs rest) | | | 0.84 | 0.4 | 1.75 | 0.64 | 2.2 | 1.1 | | | 4.2 | | **0.01** | |
|  | |  | | | | | | |  |  |  |  |  |  |
|  | |  | | | | | | |  |  |  |  |  |  |
|  | |  | | | | | | |  |  |  |  |  |  |
|  |  | | | | | | |  |  |  | |  | |  |
|  |  | | | | | | |  |  |  | |  | |  |
|  |  | | | | | | |  |  |  | |  | |  |
|  |  | | | | | | |  |  |  | |  | |  |
